# The Involvement of Descending Pain Inhibitory System in Electroacupuncture-Induced Analgesia

**DOI:** 10.3389/fnint.2019.00038

**Published:** 2019-08-21

**Authors:** Qiuyi Lv, Fengzhi Wu, Xiulun Gan, Xueqin Yang, Ling Zhou, Jie Chen, Yinjia He, Rong Zhang, Bixiu Zhu, Lanying Liu

**Affiliations:** ^1^School of Acupuncture-Moxibustion and Tuina, Beijing University of Chinese Medicine, Beijing, China; ^2^Journal Center of Beijing University of Chinese Medicine, Beijing University of Chinese Medicine, Beijing, China; ^3^School of Traditional Chinese Medicine, Beijing University of Chinese Medicine, Beijing, China; ^4^Department of Nephrology, Beijing University of Chinese Medicine Third Affiliated Hospital, Beijing, China

**Keywords:** electroacupuncture, analgesia, descending pain inhibitory system, serotonin (5-HT), noradrenaline (NA)

## Abstract

Chronic pain is a major health problem, which can impair quality of life and reduce productivity. Electroacupuncture (EA), a modality of medicine based on the theories of Traditional Chinese Medicine (TCM), presents great therapeutic effects on chronic pain. Its clinical application has gained increasing popularity, and in parallel, more research has been performed on the mechanisms of EA-induced analgesia. The past decades have seen enormous advances both in neuronal circuitry of needle-insertion and in its molecular mechanism. EA may block pain by activating the descending pain inhibitory system, which originates in the brainstem and terminates at the spinal cord. This review article synthesizes corresponding studies to elucidate how EA alleviate pain *via* the mediation of this descending system. Much emphasis has been put on the implication of descending serotonergic and noradrenergic pathways in the process of pain modulation. Also, other important transmitters and supraspinal regions related to analgesic effects of EA have been demonstrated. Finally, it should be noticed that there exist some shortcomings involved in the animal experimental designed for EA, which account for conflicting results obtained by different studies.

## Introduction

Acupuncture has been practiced as a prevalent methodology of treating human diseases in China for more than thousands of years. Among its therapies, the marked effect of relieving pain has achieved widespread acceptance, particularly chronic pain. Chronic pain is defined as pain that lasts for more than 3 months. It is a main public health problem that would impair the quality of life and is always companied by negative emotions (Treede et al., 2019). The application of acupuncture represents a satisfactory therapeutic strategy to relieve pain. To date, acupuncture has gained popularity in many countries, with the World Health Organization (WHO) deciding to maintain official relations with the World Federation of Acupuncture-Moxibustion Societies (WFAS) in the early of this year.

For a long period of time, Chinese acupuncturists tended to use the theory of Traditional Chinese Medicine (TCM) to lead clinic decisions. According to the traditional theory, acupuncture exerts its magical effect through dredging the meridian. There are a total of 20 meridians consisting of 361 acupoints traveling along the body and it is crucial to select special acupoints matched with symptoms to stimulate. The insertion and rotation of needle at specific acupoints can activate the meridians to regulate Qi. Since Qi-stagnation in the meridians is the root of pain and other diseases, an important evaluation index of acupuncture is Deqi (the getting of Qi): a sensation of heaviness, soreness or numbness during the therapy. Generally, a stronger feeling of Deqi is an implication of a better therapeutic effect.

However, the traditional theory has many limitations because modern scientific technologies have yet to decipher or detect the nature of acupoints or meridians. Though the acupuncture-induced analgesia has been confirmed by many clinical experiences, this therapeutic effect has long been questioned to be the result of hypnosis or placebo. To certify the scientific basis of acupuncture, many efforts have been taken to figure out the biological and physiological theories (Zhao, 2008; Leung, 2012; Kim W. et al., 2013; Yu et al., 2013). In the past decades, noteworthy progress has been seen in exploring the underlying mechanisms, especially the areas of neuroscience concerning that the description of meridians is similar to the neural circuit. For example, 20 years of research conducted by Pomeranz and Berman (2003) led to an opinion that acupuncture stimulation will send signals to the spinal cord and then three regions, spinal cord, midbrain and pituitary will be triggered to release corresponding transmitters to relieve pain. As a pioneer in the field of modern acupuncture, Han et al. (1985) and Han (2003, 2011) found the relationship between acupuncture analgesia and the release of opioid peptides in the nerve system.

Compared to traditional manual acupuncture (MA), the application of electroacupuncture (EA) is more suitable for scientific research, because EA can be standardized by parameters, including frequency, intensity and duration et cetera. Considering the difference between MA and EA, this review article only refers to the experimental findings using EA manipulation. In animal studies, rodents are widely used in preclinical research. The two common pain models are inflammatory pain model, such as Complete Freund’s adjuvant (CFA) induced inflammation, and neuropathic pain models, such as chronic constriction injury (CCI). After the establishment of pain models, EA stimulation will be conducted, according to traditional theory or clinical experience. By attaching battery-operated electronic devices to the needles, EA could run a mild electrical current *via* acupoints, causing the muscles to twitch. The applications of nerve severance, focal lesion of nerve and focal injection of transmitter blockers or agonists are mature methods to explore the mechanisms of EA analgesia. Behavior test is an important index to assess the threshold of pain, thus it can be used to evaluate the analgesic efficacy of EA. The paw withdrawal threshold (PWT), tail withdrawal latency (TWL) and tail flick latency (TFL) are frequently-used behavior tests.

Since the 1970s, there has been an accumulating preliminary evidence-based research from human and animal studies that describes a neural mechanism for EA-induced analgesia (Cheng, 2014; Lai et al., 2019). Early works have demonstrated the vital role of endogenous opioid in the effect of acupuncture. Subsequently, the participation of more neurotransmitters and their receptors has also been proven. It is acknowledged that EA stimulation can send signals from peripheral sites to the central regions where the sensory information will be integrated (Hu, 1979; Kawakita and Funakoshi, 1982; Toda, 2002). Moreover, the increasing awareness of the important role of descending pain inhibitory system in modulating pain has drawn increasing attention to the connection between this system and EA effect. This review is aimed at a better understanding of the intimate involvement of descending pain inhibitory pathway in EA-induced analgesia, although no consensus has been reached. The apprehension of this linkage between peripheral EA stimulation and descending pathways can not only elucidate the central mechanism of EA but also possibly lead significant therapeutic promise as it translates from bench to bedside.

## An Overview of Descending Pain Inhibitory System

Pain is not a hard-wired system that purely depends on the ascending pain pathway transmitting noxious inputs to the brain. However, the ascending nociceptive signal can be regulated by the descending system, which originates in the brainstem and terminates in the spinal dorsal horn (SDH; Sandkühler, 1996; Pertovaara and Almeida, 2006). It is widely accepted that the effect of descending system can be either inhibitory or facilitatory (Wall, 1967; Porreca et al., 2002). Compelling evidence suggests that pain is associated with the malfunction of descending pain modulation: the imbalance of the descending system to enhance facilitation or diminish inhibition may exacerbate the chronic pain condition. For example, after a nerve injury, a dysregulation in descending pathway, leading to a loss of inhibition, plays an important part in the development of neuropathic pain (Basbaum et al., 2009). It is considered that in inflammation models, the dominance of descending inhibition could attenuate the primary pain (primary hyperalgesia or allodynia), while the dominance of descending facilitation could promote the secondary pain (secondary hyperalgesia or allodynia; Vanegas and Schaible, 2004). Although pain facilitatory effects may also be related to EA treatment, in this review article, we will focus only on the underlying mechanisms of how the descending pain inhibitory system mediates the analgesic effect of EA.

The spinal cord is regarded as an important site for information integration and its hyperexcitability could be influenced by the descending inhibitory pathways. The descending inhibitory pathways project along the dorsolateral funiculi (DLF) onto the SDH and have connections with pain-related neurons in the SDH, including the terminals of primary afferent fibers, projection neurons, excitatory interneurons, inhibitory interneurons, and the terminals of other descending pathways (Millan, 2002). The nociceptive inputs from the peripheral sites ascending through the ventral part of the spinal could trigger the supraspinal regions to activate the descending inhibitory system *via* the DLF which in turn reduces the activities in pain processing (Saadé et al., 2014). The lesion of DLF blocks EA-induced analgesia while the electrical stimulation of DLF decreases the release of c-Fos expression in the SDH (Zhang et al., 1994; Li et al., 2007). The results suggest that the descending inhibitory pathways, which conduct information *via* the DLF, play an important role in the analgesic effect of EA.

The well-known descending pain inhibitory system arises in the brainstem. The periaqueductal gray (PAG) was first demonstrated as an important site where the direct injection of opioids or electrical stimulation permitted surgery in the absence of general anesthesia, which means the PAG could produce naloxone-reversible analgesia (Yaksh and Rudy, 1977; Apkarian et al., 2009). As the source of descending inhibitory pathways, the PAG does not project to the SDH directly, but has reciprocal contacts with the rostroventromedial medulla (RVM), which is considered as the final common relay for descending inhibitory pathways. The nociceptive signal from the peripheral tissues is sent to the SDH, giving rise to a hyperexcitation of ascending pathways which terminate at the PAG, RVM and other brain nuclei (Boadas-Vaello et al., 2016). The changes of transmitters occurring in the PAG and RVM will, in turn, lead to an enhancement of descending inhibition. The PAG and RVM also receive the input from higher brain regions involved in the descending pain circuits. Much attention has been paid to the anterior cingulate cortex (ACC), hypothalamus, nucleus tractus solitarii (NTS), dorsal reticular nucleus (DRt) and caudal lateral ventrolateral (VLM) et cetera. Undoubtedly, it is logical to postulate that EA-induced analgesia implies more intricate interactions of multiple supraspinal sites and that the inhibitory effects of EA are connected closely to the supraspinal modulation pain mechanism (Liu et al., 1986).

The significant transmitters released by descending inhibitory pathways involve serotonin (5-HT) and noradrenaline (NA), and there are two major monosynaptic descending pathways recognized as descending serotonergic and noradrenergic pathways. The descending serotonergic pathways originate from the nucleus raphe magnus (NRM), which is one of the rich-5-HT nuclei (Millan, 2002; Boadas-Vaello et al., 2016). The locus coeruleus (LC) which can release the NA into the SDH, constitutes the source of descending noradrenergic pathways (Jones, 1991). It has been demonstrated that EA inhibits hyperalgesia by activating supraspinal 5-HT-containing nuclei NRM and norepinephrine-containing nuclei LC, both of which terminate at the spinal cord (Li et al., 2007). Both spinal and supraspinal monoamines, 5-HT and NA, play a pivotal role in mediating EA-induced analgesia. Thus, it is also of high interest to investigate the interactions between subtypes of serotonergic and noradrenergic receptors. The details are within the scope of the present review article.

## Descending Serotonergic Pain Inhibitory Pathways

Cheng and Pomeranz (1979) reported that EA could induce parachlorophenylalanine (a 5-HT synthesis inhibitor) reversible analgesia at high-frequency (100 Hz) stimulation, resulting in an articulation that 5-HT may be partly involved in EA treatment. Of particular note is that serotonergic receptor has multiple subtypes in the nerve system (Millan, 2002). To clarify whether analgesic effects of EA depend on the different receptor types, much effort was devoted to observing the analgesic effects of combining the applications of receptor-specific antagonists or agonists with EA manipulation. Seo et al. (2016) conducted studies to observe the analgesic effects of the EA at ST36 (Zusanli) in the osteoarthritis (OA) rat model with the pretreatment of intraperitoneal injection (i.p.) of the serotonergic receptor agonists or antagonists. The results showed that EA-induced analgesia could be attenuated by 5-HT1 and 5-HT3 receptor antagonists but not by 5-HT2 receptor antagonists. This conclusion is consistent with some previous studies indicating that 5-HT1 and 5-HT3 receptors are involved in EA inhibition of chronic pain when the corresponding receptor antagonists were administrated intraperitoneally (i.p.; Baek et al., 2005; Yoo et al., 2011). However, inasmuch as those drugs were injected systemically (i.p.), it’s hard to figure out whether 5-HT exerts inhibitory influence through descending pathway or ascending pathway, and the level of neural axis at which the 5-HT takes effect.

A hypothesis was put forward: descending serotonergic pathway may not be as important as the ascending pathway in the action of EA-induced analgesia (Pomeranz and Berman, 2003). This hypothesis is in line with previous studies which lead to a conclusion that a reduction of 5-HT content in the SDH does not influence the EA effects while a decrease of 5-HT content in the forebrain impedes the analgesic effect of EA (Han et al., 1979). The results may underline the role of ascending but not descending serotonergic pathways in EA treatment. Adversely, Takagi et al. (1996) supposed that EA may activate the descending serotonergic pathways to inhibit the release of substance P whereby produce significant analgesia. Up to now, compelling evidence supports the idea that descending serotonergic inhibitory modulation also play a crucial role in the EA treatment whereas the aforesaid standpoint that the ascending pathways predominate over descending pathways may be no more persuasive.

### Supraspinal Levels

The 5-HT-containing neurons found in the medulla include the NRM, which is one of the origins of descending serotonergic inhibitory pathways. The lesions of NRM attenuate the morphine-induced analgesia and contribute to a decreased nociceptive threshold (Proudfit, 1980). This pathways participant in the pain modulation process at the spinal level *via* the DLF (Saadé et al., 2014). DLF lesions were found to emulate the consequence of spinal cord injury and block the antinociceptive effects mediated by the NRM (Xiang et al., 1991; Huang and Grau, 2018). Liu et al. (1986) reported that the inhibitory effects of EA would be reduced or eliminated after the transection of DLF and EA would increase the firing rates of neurons in the NRM to suppress the nociceptive responses. It was verified by the studies using glass microelectrode to record neuron responses in the NRM (Liu, 1996). The EA stimulation at ST36 (Zusanli) could trigger the NRM neurons to produce the analgesic effects and the activation of NRM is also modulated by higher brain regions, the PAG, for instance. Moreover, the release of 5-HT in the SDH can be evoked by microinjection of morphine into the PAG (Yaksh and Tyce, 1979). Thus, it is obvious that the EA signals can activate the NRM, from which the information descend *via* DLF to the SDH, thereby blocking the nociceptive input from the primary afferents.

Generally, the intracerebroventricular injection (i.c.v.) of the corresponding agonists or antagonists is regarded as a common method to investigate the effect of specific receptors in the CNS because the drugs could bypass the blood-brain barrier. Chang et al. (2004) demonstrated the involvement of 5-HT1A and 5-HT3 in the actions of EA-induced analgesia, but the involvement of 5-HT2A in nociceptive response when corresponding antagonists of those three serotonergic receptors were administrated intracerebroventricularly (i.c.v.). It is likely that the effects of 5-HT1A and 5-HT3 at supraspinal levels share some similarity with the situations when injected systemically (i.p. or i.v.). There exists another postulation that 5-HT may be implicated in the EA tolerance. EA tolerance is known as a universal phenomenon that repeated or long-lasting stimulation will attenuate the analgesic effect of EA. It has been revealed that the intracerebroventricularly injection (i.c.v.) of 5-hydroxytryptophan (the precursor of 5-HT) was found to reverse the EA tolerance (Li et al., 1982). Moreover, Tang et al. (1981) reported that prolonged EA stimulation would lead to a tolerance to the profound release of 5-HT, which in turn constitutes the EA tolerance.

### Spinal Cord Level

There is accumulating evidence indicating that 5-HT mediates the EA analgesia spinally and the analgesic effects depend on the different serotonergic receptor subtypes. Among the subtypes (5-HT1–7), more attention has been paid to the effects of 5-HT1, 5-HT2 and 5-HT3 on the processing of pain modulation at the spinal level (Eide and Hole, 1991).

Kim et al. (2005b) conducted studies to examine the antagonism effect of 5-HT3, 5-HT1A and 5-HT2A receptor antagonists (i.t.) on EA-induced anti-allodynia in neuropathic pain rat model (after nerve injury). The experimental results showed that spinal 5-HT1A and 5-HT3 rather than 5-HT2A mediated the analgesic effects of EA. Similar but not completely consistent results have been achieved in oxaliplatin-induced neuropathic cold allodynia rat model (Lee et al., 2016). This study was performed to observe the pain-relieving effects of EA combined with the injection intrathecally (i.t.) of 5-HT3, 5-HT1A and 5-HT2A receptor antagonists respectively by measuring the withdrawal latency. The results demonstrated that EA could alleviate the neuropathic pain *via* 5-HT3 receptors but not 5-HT1A or 5-HT2A receptors. Collectively, the involvement of 5-HT3 receptors in EA-induced analgesia is readily ascertainable while the role of 5-HT1 is controversial. It should be noted that the two studies mentioned above used two different methods to model neuropathic pain and it could help explain the discrepancies, considering that the peripheral nerve injury and the injection of oxaliplatin may have somewhat different mechanisms to induce the neuropathic pain.

However, the involvement of 5-HT1 receptors in pain modulation has been verified by many experiments. Much data demonstrated that the activation of 5-HT1 receptors may offer specific therapeutic opportunities to relieve the chronic pain (Xu et al., 1994; Bardin, 2011). Furthermore, in CFA-induced inflammatory hyperalgesia rat model, the experiments with the intrathecal injection (i.v.) of different serotonergic receptor antagonists before the EA manipulation draws the conclusion that the participation of 5-HT1A receptors in the EA-induced analgesia (Zhang et al., 2012). Similarly, another experiment showed that in inflammatory pain, the EA stimulation may facilitate the release of 5-HT that bind with 5-HT1A receptors in the SDH (Zhang et al., 2011b). Thus, it is not unreasonable to assume that in some cases, the involvement of 5-HT1A receptors is implicated in the analgesic effects of EA.

However, some studies drew different conclusions with the treatment of transcutaneous electrical nerve stimulation (TENS). TENS is another common nonpharmacologic method of pain relief by attaching the pads directly to the skin with a mild electrical current. The similarity of TENS and EA is the involvement of passage of electrical energy and the production of muscular contraction, but the difference lie in whether there is insertion of needles. Using skin electrodes to replace needle insertion, TENS is safer and more convenient. Sluka et al. (2006) reported that blockade of 5-HT2 and 5-HT3 receptors in the spinal cord reverses the analgesic effect of low-frequency TENS. Similarly, Radhakrishnan et al. (2003) showed that 5-HT2A and 5-HT3, but not 5-HT1A are implicated in TENS anti-hyperalgesia. Those results lead to a postulation that different stimulation methods (TENS vs. EA) or pain models may account for aforesaid discrepancies (Kim et al., 2005b). Moreover, Takagi and Yonehara (1998) conducted studies by intravenously administrating (i.v.) different serotonergic receptor subtypes antagonists showing that both 5-HT2A and 5-HT1A receptors in the spinal trigeminal nucleus (STN) suppressively act on EA-induced analgesia. This discordance may be due to the different research subjects (STN vs. SDH), assuming that the effects of serotonergic receptors differ from the STN and SDH, or due to the different administrative maneuvers (i.v. vs. i.t.) because systemic injection (i.v.) of the antagonists cannot demonstrate their specific actions on the spinal cord.

## Descending Noradrenergic Pain Inhibitory Pathways

In analogy to 5-HT, increasing studies are showing the crucial role of NA in the modulation of pain. NA is a potent inducer of analgesia through the activation of noradrenergic pathways. The systemic injection (i.p.) of NA receptor antagonists could block the analgesic effect of EA (Park et al., 2012). Prior to ascending pathways, the noradrenergic pathways descending to the SDH have a critical influence on the modulation of nociceptive information (Millan, 2002). Originating in the brainstem, the descending noradrenergic pathways can be activated by EA manipulation through the DLF (Hu et al., 2016). Inasmuch as the NA exerts actions at many levels of neural axis, more specific effects of NE and its descending pathways on the EA intervention will be clarified in the following paragraphs.

### Supraspinal Level

Previously, Kim H. Y. et al. (2013) synthesized several experiments into a concept that the EA stimulation may share similarities with the activation of noradrenergic cell groups in the brainstem, which produces potent analgesic effects. In the brainstem, the noradrenergic cell clusters are localized in the A1–A7, connected with other central nucleus of PAG, amygdala, preoptic area, paraventricular nucleus of the hypothalamus and lateral hypothalamus (Bajic and Proudfit, 1999). Among them, the neurons in the A5, A6 and A7 projects onto the SDH and constitute the sources of descending noradrenergic inhibitory pathways. The A6, also known as the LC, is the largest cluster of noradrenergic neurons and plays an important role in autonomic regulation of neurons in the forebrain structures, the brainstem and the SDH (Cedarbaum and Aghajanian, 1978). Due to its morphological and anatomical significance, the LC is worthy of being paid more attention to the role in EA anti-nociception. With the double immunofluorescence staining, the manipulation of EA in the inflammatory pain rat model was found to increase the expression of c-Fos in the LC and the Fos-like immunoreactive (FLI) neurons are colocalized with catecholaminergic neurons (Li et al., 2007). The results indicate that EA can activate catecholamine-containing neurons in the LC. Furthermore, the ratio of colocalization between catecholaminergic neurons and FLI neurons depends on the different EA frequencies. At low frequency, the enhancement of neuron activities in the LC is more significant (Kwon et al., 2000). Therefore, it is plausible that the neurons in the LC exert a frequency-dependent effect on the EA intervention. Recently, the same outcomes have been achieved in studies on goats, with EA exhibiting an increasing number of c-Fos in the LC and enhances the threshold of pain which, once again, demonstrated that the LC activities can be triggered by EA stimulation (Qiu et al., 2015; Hu et al., 2016). Based on the results mentioned above, it is clear that LC, as an important part of descending noradrenergic pathway, is implicated in EA-induced analgesia.

### Spinal Cord Level

A similar finding was acquired by using an *in vivo* extracellular single-unit recording, in which EA reduces the neuron activities of SDH through the activation of *α*-adrenoreceptor (Kim et al., 2011). Among the diversity of *α*-adrenoreceptors in the DH, *α*2-adrenoceptors are specifically involved in mediating the antinociception produced by *α*-adrenoceptor agonist (Yaksh, 1985; Millan, 1997), whereas *α*1-adrenoceptors appear to be in pain facilitation rather than pain inhibition (Holden et al., 1999).

A series of studies have been conducted to distinguish the role of *α*1- and *α*2-adrenoceptors in EA-induced analgesia. Kim et al. (2005a) reported that *α*2-adrenoceptors exert an inhibitory influence on neuropathic pain while *α*1-adrenoceptors exacerbate it. On this basis, Kim et al. (2005b) conducted another research combined with EA insertion. In a neuropathic pain rat model, *α*1- and *α*2-adrenoceptor antagonists (prazosin and yohimbine respectively) were injected intrathecally and afterwards EA stimulation was delivered to ST36 (Zusanli). The EA-induced anti-allodynia was reversed by the *α*2- but not the *α*1-adrenoceptors antagonists. Consistent with this result, Koo et al. (2008) reported that in an ankle sprain pain rat model, intrathecal administration of *α*2-adrenoceptor antagonists (yohimbine) reduced the analgesic effect of EA but *α*1-adrenoceptor antagonists (terazosin) did not. Collectively, these studies lead to a notion that EA-induced analgesia is mediated by *α*2-adrenoceptors at a spinal level. Furthermore, the location of *α*2-adrenoceptors is found on primary afferent by using immunohistochemical staining (Zhang et al., 2012). The activation of *α*2-adrenoceptors by EA stimulation could prevent the phosphorylation of the NMDA receptor (NMDAR) NR2 subunits (pNR2B; Choi et al., 2015). NMDAR is known as the receptor of glutamate and the pNR2B could maintain the neuropathic pain powerfully. Taken together, it is rational to assume that EA may increase the release of NA binging to *α*2-adrenoceptors and thus lead to post-synaptically inhibition of pNR2B to relieve pain. But this postulation requires further experimental investigation. To examine the distinction among the subtypes of *α*2-adrenoceptors, Zhang et al. (2012) conducted research administering subtype selective agents, *α2a-* and *α*2b-adrenoceptors antagonist respectively (i.t.) before EA stimulation in a CFA-induced inflammatory pain. The data showed that *α*2a-adrenoceptors are involved in the EA inhibition, while *α*2b-adrenoceptors have no effect. At spinal level, the opioid-containing terminals coexist with the adrenergic *α*2c-adrenoceptors and the activation of *α2c-adrenoceptors* may suppress the actions exerted by opioids (Chen et al., 2008). Considering that the opioidergic system underlies the mechanisms of EA analgesic effect, the mediation of *α2c-adrenoceptors* in EA effect is possible.

There exists a kind of chemical acupuncture called apipuncture. The operation of apipuncture is subcutaneous administration (s.c.) of diluted bee venom (DBV) into specific acupoints. Bee venom is a complex mixture extracted from honeybee and therein various peptides exert anti-inflammatory antioxidant and anti-apoptosis properties. The combination of traditional acupuncture and DBV has been widely used in rheumatoid arthritis (RA), neurodegenerative diseases, liver disorder, cardiovascular diseases (Zhang S. et al., 2018). The application of apipuncure strengthens the stimulation of acupoints but the biological mechanisms responsible for its antinociceptive effects need further investigation. Several lines of evidence suggest that the apipuncture also has analgesic effects through the noradrengic system (Yoon et al., 2009). Kim et al. (2005) conducted studies with the treatment of apipuncture at ST36 (Zusanli). The apipuncture was applied to the formalin-induced inflammatory rat model after the intrathecal injection (i.v.) of antagonists of different adrenoceptor subtypes. The result revealed that the apipuncture could produce analgesic effect through the activation of the *α*2-adrenoceptor but not *α*1- or β-adrenoceptor at the spinal level. This is verified by the result that DBV-induced analgesia is mediated by both *α2a-* and *α2c-adrenoceptors* (Kang et al., 2011). In addition to the inflammatory pain, apipuncture exerts analgesic effects on visceral pain and neuropathic pain as well (Kwon et al., 2001; Roh et al., 2004). Consistently, the injection of DBV into peripheral tissues was found to increase Fos expression in the LC, which implies that apipuncture may be mediated by the activation of central noradrenergic nuclei including the LC (Kwon et al., 2006).

## Other Transmitters Related to EA-Induced Analgesia

### Cholecystokinin Octapeptide (CCK-8) and EA Tolerance

The peptide CCK-8 exerts anti-analgesic actions site- and dose-dependently in the both peripheral and central nervous systems (Rehfeld, 1980; Moran and Schwartz, 1994). It is generally accepted that CCK-8 can reverse the antinociceptive effect of morphine and therefore suppress the process of descending inhibitory initiated by opioids (Rehfeld, 1980; Lapeyre et al., 2001). Given the fact that opioids play a vital role in EA-induced analgesia, it is possible to assume the involvement of CCK-8 in reversing and prevention of EA antinociceptive effects. CCK-8, given both intracerebroventricularly (i.c.v.) and intrathecally (i.t.), blocked the EA effects in the rat (Han et al., 1985). Taking the TFL as an indication of nociception, subcutaneous (s.c.) injection of the CCK-B receptor antagonist (L-365, 260) facilitated the analgesia induced by 100 Hz EA (Huang et al., 2007). Further studies have shown that analgesic effects of EA can be facilitated by intrathecal (i.t.) administration of L-365, 260, indicating the mediation of CCK-B receptor in the effect of CCK-8 (Zhou et al., 1993). All these converge towards the conclusion that CCK-8 and CCK-B receptor synergistically exert an antagonistic effect in EA-induced analgesia *via* their negative feedback mechanism producing a reduction of released opioids endogenously (Han, 1995).

However, CCK-8 shows no effect on the EA-induced analgesia mediated by 5-HT or norepinephrine, and furthermore, CCK-8 antagonists do not enhance the baseline of nociception (Han et al., 1986). From this, Han et al. (1986) postulated that CCK-8 may participate in the process of EA tolerance, which has been verified by compelling evidence. EA tolerance terms the phenomenon that repeated and chronic stimulation of EA will impair its ability to relieve the pain (Han et al., 1981). By using single-neuron recording in a previous study, injection (i.c.v.) of CCK-8 antiserum potentiated the EA effect which otherwise would subside after prolonged (6 h) stimulation (Bian et al., 1993). Emerging evidence concerning that profound release of opioids induced by long-lasting EA stimulation could elicit up-regulation of CCK mRNA at both spinal and supraspinal levels (Zhou et al., 1992; Ding and Bayer, 1993). The spinal cord is regarded as an important site (Wiesenfeld-Hallin and Xu, 1996), whereas it is also supported that nucleus accumben is a strategic area for CCK-8 and CCK-B receptors to exert anti-opioid effects (Pu et al., 1994).

It should be noted that CCK-8 does not exert the same anti-opioid actions towards the different conditions. The release of CCK-8 can be more likely increased by high-frequency (100 Hz) than low-frequency EA (15 or 2 Hz; Chen et al., 1994). Among the subtypes of opioid receptor, antinociceptive effects mediated by k- and μ-receptor can be blocked by CCK but δ-receptor makes very little difference (Wang et al., 1990; Benoliel et al., 1994; Noble et al., 1995). Moreover, individual sensitivity to acupuncture can influence the distribution and release of CCK (Tang et al., 1997; Zhao, 2008).

In addition to CCK-8 antiserum and CCK receptor antagonist, there are other matters associated with EA tolerance. Administration of ketamine (i.p.) can prevent or delay the development of EA tolerance through peripheral NMDARs, and similarly, nocistatin (i.c.v) is involved in reversing chronic tolerance to EA antinociception *via* cerebral mechanisms (Huang et al., 2004, 2005). Those results may provide potential opportunities to improve the therapeutic effect of EA when tolerance occurs.

### Opioidergic Mechanisms in the PAG

One of the most pioneering findings of neurobiology mechanisms underlying the EA effects is that EA could produce partially naloxone-reversible analgesia (He and Dong, 1983). This demonstrated the involvement of endogenous opioidergic system in the EA-induced analgesia. The opioid peptides, including endomorphin, dynorphin, enkephalin and β-endorphin, bind to their receptors and exert analgesic effects. Following EA stimulation, the expression of opioid peptides may be upregulated.

Endogenous opioids can mediate the effect of EA at almost all neural axis. However, in this review article, we only focus on the opioidergic mechanisms in the PAG. As a vital integrative center in antinociceptive effect of EA, the PAG receives not only the ascending nociceptive input but also the descending anti-nociceptive signals from the higher central regions. The RNA sequencing performed in goat PAG showed that five neuropeptides genes, glutamatergic synapse, GABAergic synapse, MAPK, ribosome and ubiquitin-proteasome pathway may be implicated in EA-induced analgesia (Hu et al., 2017).

Xuan et al. (1986) have proposed a concept known as a “mesolimbic neural loop” through which many brain nuclei including the PAG, nucleus accumbens, habenula and amygdala are involved in analgesic effects. In this neural loop, opioid peptides are involved and the PAG is a strategic area. Previous studies demonstrated that microinjection of morphine into PAG could increase the release of enkephalin and β-endorphin in the nucleus accumbens and* vice versa*, which in turn could produce naloxone-reversible anti-nociception (Ma and Han, 1991; Han and Xuan, 2009). Compelling evidence indicates that EA and opioids share similarities in analgesia and the release of opioid peptides in the PAG has been proven to be modulated by EA stimulation (He and Han, 1990; Niddam et al., 2007). He and Dong (1983) has conducted experiments using multi-micropipettes for extracellular recording and the results have led to a postulation that EA stimulation may activate the opioidergic system in the PAG to inhibit the spontaneous neuronic discharge and the conveyance of nociceptive signals. Among the opioid peptides, the enkephalin and β-endorphin contribute much more to the analgesic effect of EA in the PAG. The EA treatment could activate neurons in the vlPAG and the activated neurons would interact with nerve fibers containing enkephalin or β-endorphin in the vlPAG (Guo et al., 2004). Consistently, an injection of anti-metenkephalin serum (AMEKS) and anti-β-endorphin serum (AEPS) into the PAG was found to attenuate the potentiation effect of neurotensin (NT) on EA-induced analgesia, which demonstrates a synergetic relationship among enkephalin, β-endorphin and NT (Bai et al., 1999).

As a newly-discovered neuropeptide, Orphanin FQ (OFQ) is in the family of endogenous opioid peptide but has different physiological functions (Winters et al., 2019). OFQ immunoreactivity could be observed in the ventrolateral PAG (vlPAG), NRM, dorsal raphe nucleus (DRN; Ma et al., 2004). The experimental results obtained in rats showed that intrathecally injection (i.t.) of OFQ exerted synergetic effects with EA whereas intracerebroventricular injection (i.c.v.) of OFQ produced antagonism of analgesia induced by EA (Tian et al., 1997). These findings suggest that spinal OFQ may potentiate the EA-induced analgesia, which was verified by the subsequent research (Fu et al., 2006), whereas OFQ in the brain nuclei may antagonize it. This conclusion is in congruence with previous studies indicating that OFQ in the PAG plays a role in hyperalgesia and has dose-related antagonization on EA effect (Wang et al., 1998). Consistently, Zhou et al. reported that reduction of release and synthesis of OFQ in the brain nuclei may underlie the mechanisms of synergetic effect of melatonin on EA analgesia (Zhou et al., 2001). Collectively, all these converge towards the conclusion that in the PAG, OFQ and other opioid peptides show an anti-action on EA-induced analgesia.

### GABAergic System at Spinal and Supraspinal Level

EA could produce analgesia by increasing the release of inhibitory amino acids and decreasing the release of excitatory amino acids (Yan et al., 2011). γ-aminobutyric acid (GABA) is a vital inhibitory amino acids and GABAergic neurons are considered as an important link in the descending inhibitory pathway. There exists a postulation that endocannabinoid (2-AG)-cannabinoid receptor (CB1R)-GABA-5-HT may be a novel signaling pathway in the EA-induced analgesia (Yuan et al., 2018).

There are many subtypes of GABA receptors and therein GABA-A and GABA-B receptors are revealed to be implicated in pain modulation. Previous studies have well-documented that the pain threshold of tail neuropathic pain could be increased by EA stimulation through the upregulation of GABA expression spinally and by taking tail immersion test as an indication of nociception, the results showed that anti-allodynia effect of EA could be blocked by intrathecal administration (i.t.) of gabazine and saclofen (GABA-A and GABA-B receptor antagonists respectively). The findings demonstrated the involvement of GABA receptor subsets GABA-A and GABA-B receptors in EA-induced analgesia at the spinal level (Park et al., 2010). This conclusion has been verified by studies of inflammatory pain rat, which observed the upregulation of GABA-A and HABA-B receptors mRNA in the cervical spinal cord after the treatment of EA (Gao et al., 2012). Hence, those are in accordance with the recent results indicating that the expression of GABA-A receptors in the spinal cord could be increased by EA to relieve neuropathic pain (Jiang et al., 2018). Therefore, sufficient evidence exists to indicate that both GABA-A and GABA-B at spinal level are implicated in EA-induced analgesia. In addition, the increased levels of GABA-A and GABA-B receptors may downregulate the activities of substance P (SP) neurons and satellite glial cells (SGCs) and in turn fulfill analgesic effects (Qiao et al., 2017, 2019).

GABAergic system also plays an important role in the EA effect at the supraspinal level. After EA stimulation, the expression of GABA was found to overlap the c-Fos expression in the PAG, which revealed that the GABAergic system in the PAG may participant in descending inhibition activated by EA (Fusumada et al., 2007). It is hypothesized that at supraspinal level, only GABA-B receptors participant in EA-induced analgesia (Zhao, 2008). The role of GABA-B in the ACC has been found to be associated in mechanical hypersensitivity and GABA-B receptors are involved in the morphine-induced and muscarinic M1 receptor-mediated analgesia (Migita et al., 2018; Pedrón et al., 2019). Hence, Zhang Q. et al. (2018) demonstrated that GABA-B receptors in the NTS may be regulated by EA manipulation. However, sensitivity bias of GABA-A and GABA-B receptors in supraspinal structures warrants further investigation and that detail is beyond the scope of the present review article.

## Other Supraspinal Regions Related to EA-Induced Analgesia

### Hypothalamus

The hypothalamus connects with the NTS, the PAG and the RVM, which are implicated in the nociceptive processing and descending pathways. Pomeranz and Berman (2003) hold the view that the descending projections from hypothalamus release β-endorphin to the midbrain and thus cause analgesia, however, the hypothalamus is only activated by low-frequency but not high-frequency stimulation. In the CCI rat model, the elevation of β-endorphin and adrenocorticotrophic hormone (ACTH) in the hypothalamus and pituitary during EA stimulation suggests the involvement of hypothalamic-pituitary axis in the analgesic effect of EA on neuropathic pain (Liu et al., 2010). Further, it should be noted that the arcuate nucleus (ARC) within the hypothalamus takes part in the process of EA treatment. Yin et al. (1988) reported that EA-induced analgesia could be enhanced by the stimulation of the ARC, indicating that ARC may participant in pain modulation of EA. It is consistent with the recent studies showing that EA at specific acupoints increased the c-Fos expressions in some hypothalamus nuclei, including the ARC and the ventromedial nucleus of the hypothalamus (VMH) in goats (Hu et al., 2016). It is also acknowledged that arcuate nucleus of the hypothalamus (HARN) is an important part of acupuncture afferent and efferent pathways (Takeshige et al., 1991, 1992a). The medial HARN (M-HARN) acts as the final sector of the afferent pathway from the acupoint, whereas the posterior HARN (P-HARN) is regarded as the origin of the efferent pathways. It is further reported that the interlinkage between the M-HARN and P-HARN may involve the transmitters, including dopamine and β-endorphin released from the pituitary by EA stimulation (Takeshige et al., 1991, 1992b).

### Nucleus Tractus Solitarii (NTS)

The NTS is a relay for the effects of vagal afferent stimulation and receives visceral nociceptive messages (Ren et al., 1990). Anatomically, the NTS connects to the hypothalamus, the cortical regions, the PAG, the brainstem and the spinal cord (Pan et al., 1999; Millan, 2002). The analgesic effects induced by electrical stimulation of the NTS indicate the relationship between the NTS and pain modulation (Lewis et al., 1987). Hence, it is not unreasonable to assume that EA exerts antinociceptive effects through the activation of the NTS. Considering that ST36 (Zusanli) is the empirical acupoint for adjusting function of gastrointestinal tract, an experiment was designed to verify the effect of EA at the ST36 (Zusanli) *via* the observation on the firing rate of gastric-related neurons in the NTS (Wang et al., 2013). The results showed that EA activated neurons in the NTS and NTS neurons presented diverse responsive modes. In accordance with the result, the study of colorectal distension (CRD) rats suggested that EA could inhibit CRD excitatory neurons in the NTS to relieve visceral pain (Liu et al., 2014). Taking extracellular recording methods as an assessment of firing neurons, the experiment demonstrated that at different acupoints, the responses of gastric distention-related neurons in the NTS are different (He et al., 2006). Moreover, Guo and Malik (2019) reported that EA at specific acupoints activates the NTS neurons which project to the rostral ventrolateral medulla (rVLM) and therefore triggers the activity of the rVLM. Collectively, the NTS may play a vital role in the EA-induced analgesia, especially for the visceral pain.

### Anterior Cingulate Cortex (ACC)

Several lines of evidence support the postulation that the descending inhibitory pathways may associate with the ACC, which connects to many pain modulation-related brain regions (Wyss and Sripanidkulchai, 1984; Ikeda et al., 2014). Anatomically, the PAG is innervated by the fibers from the ACC and the ACC may participant in PAG-RVM pathways (Tsuda et al., 2017). Further, AAC converges emotional and nociceptive information and has interaction between pain and emotional changes (Rainville et al., 1997; Johansen et al., 2001). Inasmuch as the rostral ACC (rACC) is related to negative emotions evoked by pain perception, it is not unreasonable to assume that the rACC can mediate the EA analgesia. With the study of rats injected by formalin, Yi et al. (2011) proposed some potential underlying mechanisms of how ACC mediates the analgesic effect of EA. On a short-term level, the activation of ACC may regulate the descending pain inhibitory system, whereas on a long-term level, it may trigger the affection-related regions and thereby pain-related aversiveness may be inhibited. These findings are consistent with previous reports that the connection between ACC and PAG could be inhibited by EA and thus cause disinhibition of PAG neurons that contribute to anti-nociception, which in turn activates the descending inhibitory pathways (Hirano et al., 2008). However, the relevance of ACC to acupuncture analgesia seems to be controversial with the demonstration that ACC contributes to the descending facilitatory rather than inhibitory modulation of pain (Calejesan et al., 2000; Lei et al., 2004; Zhang et al., 2005). Chen et al. (2014) reported that the activation of the cells in the ACC projecting to the DH accounts for the expression of long-term potentiation (LTP) in neuropathic pain. Furthermore, Zhao (2008) supports the idea that EA may deactivate the ACC according to the study of functional MR imaging (Wu et al., 1999). Taken together, the relationship between ACC and EA remains far from being clarified and the incompatible postulations mentioned above require further experimental investigation.

### Rostroventromedial Medulla (RVM)

The RVM contains the NRM, the nucleus reticularis gigantocellularis-pars alpha and the nucleus paragigantocellularis lateralis (Vanegas and Schaible, 2004). As a final relay in the descending pathways, the RVM receives signals from higher regions, including the PAG, the thalamus, the parabrachial region, and projects the signals to the SDH (Gebhart, 2004; Vanegas and Schaible, 2004; Song et al., 2013). The previous work has been designed to observe the expression of the c-Fos in the brainstem after EA manipulation, using double-labeling immunocytochemistry (He et al., 1992). The finding demonstrated the co-localization of c-Fos protein and 5-HT in the RVM and the critical role of RVM in the antinociceptive effect of EA. This is congruent with the notion that descending serotonergic pathways can be activated by the stimulation of descending projections from the RVM. The neurons in the RVM are distinguished by their different functional characteristics revealed by electrophysiological recordings in the RVM, and therein on-cells and off-cells are responsible for the descending facilitation and inhibition respectively (Fields et al., 1995; Ellrich et al., 2000). The off-cells induce the descending inhibition through opioid mechanisms and both 2 Hz and 20 Hz EA may activate the off cells to suppress nociceptive transmission (Heinricher et al., 1994). Furthermore, there are serotonergic and GABAergic neurons classified as primary cells and secondary cells respectively. EA may trigger the primary or secondary cell to fulfill the analgesic effects. To investigate under which mechanisms EA exerts an anti-hyperalgesia effect *via* the RVM, the studies have been conducted by injecting of μ-receptor antagonists into the RVM to observe paw withdrawal latency (PWL) of inflammatory rats after EA stimulation (Zhang et al., 2011a). The result showed the important role of intra-RVM μ-receptor in mediating the EA effects. The results from a further study that uses double immunofluorescence staining revealed that μ-receptors are located in the GABAergic neurons and corresponding GABA-A receptors are located in the serotonergic neurons in the RVM (Zhang et al., 2011a). There are reasons to believe that EA may activate the μ-receptor in the RVM by endogenous endomorphins to decrease the release of GABA and enhance the serotonergic neurons, and therefore attenuate through descending serotonergic pathways.

Besides the above, other brain regions including amygdala and parabrachial nucleus (PBN), are also involved in processing EA analgesia. The amygdala is a locus of analgesic action and engages mechanisms of descending inhibition *via* a relay in the PAG (Millan, 2002). It is generally accepted that repeated EA stimulation will activate the amygdala to relieve both sensory and affective dimensions of pain *via* up-regulation of GABA and NMDA (Duanmu et al., 2017). The PBN, situated in the brainstem, participates the process of emotional and cognitive dimensions of pain (Fields and Basbaum, 1999). The PBN receives nociceptive information from SDH and later projects to the superficial SDH laminae. Han and Wang (1991) first suggested that signals induced by high-frequency (100 Hz) of EA reach PBN which innervates vlPAG whereby descending pathways originated to release dynorphins in the SDH.

## Discussion

As an external medical therapy of TCM, EA has been accepted to treat a wide spectrum of diseases. Among its therapies, the treatment of chronic pain is in the ascendant. The interest in the biological mechanisms responsible for EA-induced analgesia has been increasing and much effort has been devoted to conduct animal experiments. However, the accurate underlying mechanisms of EA are not fully understood. This review article mainly focuses on the involvement of descending pain inhibitory system in EA-induced analgesia. This system consists of two major monosynaptic descending pathways called descending serotonergic and noradrenergic pathways. Both 5-HT and NA mediate the analgesic effects of EA manipulation in the spinal cord and higher brain regions (NRM and LC respectively) and interactions between subtypes of their receptors have also been discussed. Also, there exist other transmitters involved in descending pathways. CCK-8 contributes to EA tolerance mediated by opioids and it exerts different anti-opioid actions towards the different conditions. Opioids, enkephalin and β-endorphin especially, in the PAG facilitate the EA efficacy but therein OFQ has opposite physiological functions. Moreover, as a vital inhibitory amino-acid, GABA plays a vital role in EA anti-nociception through GABA-A and GABA-B receptors. Furthermore, we discuss some EA-related supraspinal regions omitted in the previous paragraphs. Sufficient evidence exists to demonstrate that the hypothalamus, NTS, ACC, RVM are implicated in EA-induced analgesia but additional details of underlying mechanisms require further experimental investigations.

Although studies obtained by this review article elucidate the relationship of EA-induced analgesia and descending pain inhibitory system, we notice that there are many conflicting results obtained by extensive studies. Those controversies may probably be attributed to the design of animal experiments, suggesting current research has some shortcomings that are yet to be solved. Therefore, several challenges in designing an experiment of EA-induced analgesia will be listed in the following paragraphs.

### To Remove the Interference of Stress-Induced Analgesia (SIA)

To reveal the mechanisms of EA, animals, rodents chiefly, are widely used in preclinical research. It is reasonable to postulate that EA-induced short-term analgesia may be due to the mechanisms behind stress-induced analgesia (SIA). Although the EA-induced analgesic effects share some similarities with stressful stimuli, a detailed comparison shows the patterns of c-Fos expression are different in locations (Guo et al., 1996). In many traditional studies, cylindrical restraint should be applied to rodents for EA stimulation. However, compelling evidence indicate that stress-like responses will be evoked under restraint in rodents (Zhang et al., 2013). Therefore, the effect of EA may be masked by SIA. In 2004, a novel unrestrained method was introduced, and it was verified that unrestrained rats present less stress-like behaviors after receiving EA stimulation (Lao et al., 2004; Zhang et al., 2013). Undoubtedly, this opens a novel way for further experimental designs. In addition, the unfamiliar environment may also evoke as stress response. Thus, it is important to ensure animals are accustomed to the experimental circumstances to relieve or even eliminate stress to remove disturbances.

### To Determine a Quantifiable EA Stimulation

According to the traditional theory of Chinese medicine, the manipulation of MA (including the operation of tonifying/dissipating, thrust/rotation, warming/cooling etc.) is complex. Based on different body conditions, the manipulation will be different. Thanks to the introduction of EA, the standard of parameters can be easily set and controlled, which makes EA experiments highly reproducible. However, stimulation criteria involve the stimulation frequency, duration, amplitude, intensity and so on. Compelling evidence suggests that different parameters of EA stimulation produce different analgesic effects.

Much effort was devoted to figure out how different frequencies of EA exert different analgesic effects. Silva et al. (2011) conducted studies indicating that at spinal level, 2- and 100-Hz EA exert anti-nociceptive effects through different descending mechanisms, including adrenergic, serotonergic, opioidergic, muscarinic and GABAergic systems, which is in line with a previous notion that specific frequencies could potentiate the release of specific neuropeptides (Han, 2003). Several lines of evidence suggest that opioidergic system involved in the analgesic effect is influenced by the EA frequency. For example, 2 Hz EA may favor the release of enkephalins and endorphins while 100 Hz increases the release of dynorphins (Han et al., 1999). Moreover, low and high frequencies EA may also trigger different brain regions. In anesthetized SD rats, when stimulated by 4- and 100-Hz EA respectively, the observation of the number and distribution of Fos-immunoreactive neurons suggested that different frequencies of EA analgesia could produce different distributions of c-Fos in the brain-stem and spinal cord nuclei (Lee and Beitz, 1993). Consistently, it has been reported that a 2 Hz stimulation could activate the hypothalamus, PAG, medulla and SDH whereas 100 Hz EA activates a pathway originating from the PBN (Han, 2011). It is also of interest to find out whether other parameters, including duration and intensity, also have impacts on the effect of EA. Given that prolonging the time of EA stimulation may trigger the development of EA tolerance, the duration of EA and analgesic effects are not positively correlated. Ma (2004) previously reported that 20–30 min EA treatment exhibits more efficacy and potency. It was also reported that the intensity accounts for naloxone-reversibility of EA, which means whether EA-induced analgesia is mediated by opioids depends on different EA intensity (Huang et al., 2002). Collectively, the above leads to the conclusion that EA frequency, intensity and duration all have significant impacts on EA-induced analgesia. However, the optimal EA parameters that can be applied in the clinic and experiment is far from clarified.

### To Select Specific Analgesic Acupoints

The study of functional magnetic resonance imaging (fMRI) has demonstrated that EA induces acupoint-dependent analgesia, which has been agreed by some other studies focusing on the human brain (Zhang et al., 2004; Brettmann et al., 2005; Yan et al., 2005). The same conclusion has been drawn by some animal experiments. It was previously revealed that in SD rats, the stimulation of EA at analgesic acupoints (such as LI 4) have a predominate effect when is compared with non-analgesic acupoints (Chiu et al., 2003). Similarly, Hu et al. (2016) conducted studies by using EA stimulation at two different sets of acupoints in goats to investigate analgesia neural circuits activated in the brain. The results showed that one specific set of acupoints induce higher pain threshold and the levels of c-Fos expressions by different sets of acupoints are different. All converge towards the conclusion that EA anti-nociceptive effects may have acupoint-specific difference. The method of selecting symptomatic acupoints may probably be one of the major problems impeding understanding of the EA mechanisms.

Beyond the reasons discussed above, we speculate that different results obtained by different studies may also depend on the administrative maneuver and pain models. Since nearly all levels of the neural axis contribute to the pain modulation, investigations limited to the effect of systemically administration of corresponding receptor agonist or antagonist cannot illustrate the specific sites where the action is exerted. In terms of animal models, different animal strains, types of pain and modeling methods may also be important factors in determining the underlying mechanisms of pain (Kim et al., 2005a; Silva et al., 2011). Thus, it is not surprising there exist many controversies stemming from animal experiments. Obviously, an essential underpinning of a convincing result is a high-repeatable experiment. Thus, to better understand the potential mechanisms of EA-induced analgesia, it’s imperative to establish consensus on the experimental design.

## Author Contributions

QL conceived and wrote the review article. FW, XG and XY revised the article. LZ, JC, YH, RZ and BZ analyzed the data. LL gave final approval for publication.

## Conflict of Interest Statement

The authors declare that the research was conducted in the absence of any commercial or financial relationships that could be construed as a potential conflict of interest.

## References

[B1] Apkarian A. V. Baliki M. N. Geha P. Y. (2009). Towards a theory of chronic pain. Prog. Neurobiol. 87, 81–97. 10.1016/j.pneurobio.2008.09.018 18952143 PMC2650821

[B2] Baek Y. H. Choi D. Y. Yang H. I. Park D. S. (2005). Analgesic effect of electroacupuncture on inflammatory pain in the rat model of collagen-induced arthritis: mediation by cholinergic and serotonergic receptors. Brain Res. 1057, 181–185. 10.1016/j.brainres.2005.07.014 16139820

[B3] Bai B. Wang H. Liu W. Y. Song C. Y. (1999). Effect of anti-opioid peptide sera on the enhancement of electroacupuncture analgesia induced by neurotensin in PAG of rats. Sheng Li Xue Bao 51, 224–228. 11499020

[B4] Bajic D. Proudfit H. K. (1999). Projections of neurons in the periaqueductal gray to pontine and medullary catecholamine cell groups involved in the modulation of nociception. J. Comp. Neurol. 405, 359–379. 10.1002/(sici)1096-9861(19990315)405:3<359::aid-cne6>3.3.co;2-n 10076931

[B5] Bardin L. (2011). The complex role of serotonin and 5-HT receptors in chronic pain. Behav. Pharmacol. 22, 390–404. 10.1097/fbp.0b013e328349aae4 21808193

[B6] Basbaum A. I. Bautista D. M. Scherrer G. Julius D. (2009). Cellular and molecular mechanisms of pain. Cell 139, 267–284. 10.1016/j.cell.2009.09.028 19837031 PMC2852643

[B7] Benoliel J. J. Collin E. Mauborgne A. Bourgoin S. Legrand J. C. Hamon M.. (1994). Mu and delta opioid receptors mediate opposite modulations by morphine of the spinal release of cholecystokinin-like material. Brain Res. 653, 81–91. 10.1016/0006-8993(94)90375-1 7982079

[B8] Bian J.-T. Sun M.-Z. Han J.-S. (1993). Reversal of electroacupuncture tolerance by Cck-8 antiserum: an electrophysiological study on pain-related neurons in nucleus parafascicularis of the rat. Int. J. Neurosci. 72, 15–29. 10.3109/00207459308991620 8225797

[B9] Boadas-Vaello P. Castany S. Homs J. Álvarez-Pérez B. Deulofeu M. Verdú E. (2016). Neuroplasticity of ascending and descending pathways after somatosensory system injury: reviewing knowledge to identify neuropathic pain therapeutic targets. Spinal Cord 54, 330–340. 10.1038/sc.2015.225 26754470

[B10] Brettmann K. Bürklein M. Vogt L. Banzer W. (2005). Evidence from brain imaging with fMRI supporting functional specifity of acupoints in humans. Dtsch. Z. Akupunktur 48, 54–55. 10.1078/0415-6412-00099

[B11] Calejesan A. A. Kim S. J. Zhuo M. (2000). Descending facilitatory modulation of a behavioral nociceptive response by stimulation in the adult rat anterior cingulate cortex. Eur. J. Pain 4, 83–96. 10.1053/eujp.1999.0158 10833558

[B12] Cedarbaum J. M. Aghajanian G. K. (1978). Afferent projections to the rat locus coeruleus as determined by a retrograde tracing technique. J. Comp. Neurol. 178, 1–15. 10.1002/cne.901780102 632368

[B13] Chang F.-C. Tsai H.-Y. Yu M.-C. Yi P.-L. Lin J.-G. (2004). The central serotonergic system mediates the analgesic effect of electroacupuncture on ZUSANLI (ST36) acupoints. J. Biomed. Sci. 11, 179–185. 10.1159/000076030 14966368

[B16] Chen X. H. Han J. S. Huang L. T. (1994). CCK receptor antagonist L-365,260 potentiated electroacupuncture analgesia in Wistar rats but not in audiogenic epileptic rats. Chin. Med. J. 107, 113–118. 8194376

[B14] Chen T. Koga K. Descalzi G. Qiu S. Wang J. Zhang L.-S.. (2014). Postsynaptic potentiation of corticospinal projecting neurons in the anterior cingulate cortex after nerve injury. Mol. Pain 10:33. 10.1186/1744-8069-10-33 24890933 PMC4060852

[B15] Chen W. Song B. Marvizón J. C. G. (2008). Inhibition of opioid release in the rat spinal cord by α2C adrenergic receptors. Neuropharmacology 54, 944–953. 10.1016/j.neuropharm.2008.02.002 18343461 PMC2365759

[B17] Cheng K. J. (2014). Neurobiological mechanisms of acupuncture for some common illnesses: a clinician’s perspective. J. Acupunct. Meridian Stud. 7, 105–114. 10.1016/j.jams.2013.07.008 24929454

[B18] Cheng R. S. S. Pomeranz B. (1979). Electroacupuncture analgesia could be mediated by at least two pain-relieving mechanisms; endorphin and non-endorphin systems. Life Sci. 25, 1957–1962. 10.1016/0024-3205(79)90598-8 160969

[B20] Chiu J.-H. Chung M.-S. Cheng H.-C. Yeh T.-C. Hsieh J.-C. Chang C.-Y.. (2003). Different central manifestations in response to electroacupuncture at analgesic and nonanalgesic acupoints in rats: a manganese-enhanced functional magnetic resonance imaging study. Can. J. Vet. Res. 67, 94–101. 10.1111/j.1751-0813.2003.tb12579.x 12760473 PMC227035

[B21] Choi J.-W. Kang S.-Y. Choi J.-G. Kang D.-W. Kim S.-J. Lee S. D.. (2015). Analgesic effect of electroacupuncture on paclitaxel-induced neuropathic pain *via* spinal opioidergic and adrenergic mechanisms in mice. Am. J. Chin. Med. 43, 57–70. 10.1142/S0192415X15500044 25640847

[B19] Duanmu C. L. Feng X. M. Yan Y. X. Wang J. Y. Gao Y. H. Qiao L. N. (2017). Efect of electroacupuncture intervention on expressopn of synaptic plasticity-related molecules in amdala in chronic pain-negative afection rats. Acupunct. Res. 42, 1–8. 10.13702/j.1000-0607.2017.01.001 29071990

[B22] Ding X.-Z. Bayer B. M. (1993). Increases of CCK mRNA and peptide in different brain areas following acute and chronic administration of morphine. Brain Res. 625, 139–144. 10.1016/0006-8993(93)90146-e 8242392

[B23] Eide P. K. Hole K. (1991). Different role of 5-HT1A and 5-HT2 receptors in spinal cord in the control of nociceptive responsiveness. Neuropharmacology 30, 727–731. 10.1016/0028-3908(91)90180-j 1717872

[B24] Ellrich J. Ulucan C. Schnell C. (2000). Are “neutral cells” in the rostral ventro-medial medulla subtypes of on- and off-cells? Neurosci. Res. 38, 419–423. 10.1016/s0168-0102(00)00190-5 11164568

[B26] Fields H. L. Basbaum A. I. (1999). “Central nervous system mechanisms of pain modulation,” in Textbook of Pain, 4th Edn. eds Wall P. D. Melzack R. (Edinburgh: Churchill Livingston), 309–329.

[B25] Fields H. L. Malick A. Burstein R. (1995). Dorsal horn projection targets of ON and OFF cells in the rostral ventromedial medulla. J. Neurophysiol. 74, 1742–1759. 10.1152/jn.1995.74.4.1742 8989409

[B27] Fu X. Wang Y.-Q. Wu G.-C. (2006). Involvement of nociceptin/orphanin FQ and its receptor in electroacupuncture-produced anti-hyperalgesia in rats with peripheral inflammation. Brain Res. 1078, 212–218. 10.1016/j.brainres.2006.01.026 16563360

[B28] Fusumada K. Yokoyama T. Miki T. Wang Z.-Y. Yang W. Lee N.-S.. (2007). c-Fos expression in the periaqueductal gray is induced by electroacupuncture in the rat, with possible reference to GABAergic neurons. Okajimas Folia Anat. Jpn. 84, 1–10. 10.2535/ofaj.84.1 17654838

[B29] Gao Y.-H. Chen S.-P. Wang J.-Y. Qiao L. N. Han Y.-J. Lin D.. (2012). Effects of electroacupuncture of “Futu” (LI 18), etc. on pain behavior and expression of GABA receptor subunit genes in cervical spinal cord in rats with thyroid regional pain. Zhen Ci Yan Jiu 37, 93–98. 10.13702/j.1000-0607.2012.02.005 22764592

[B30] Gebhart G. F. (2004). Descending modulation of pain. Neurosci. Biobehav. Rev. 27, 729–737. 10.1016/j.neubiorev.2003.11.008 15019423

[B32] Guo Z.-L. Malik S. (2019). Acupuncture activates a direct pathway from the nucleus tractus solitarii to the rostral ventrolateral medulla. Brain Res. 1708, 69–77. 10.1016/j.brainres.2018.12.009 30529283 PMC6378112

[B33] Guo Z.-L. Moazzami A. R. Longhurst J. C. (2004). Electroacupuncture induces c-Fos expression in the rostral ventrolateral medulla and periaqueductal gray in cats: relation to opioid containing neurons. Brain Res. 1030, 103–115. 10.1016/j.brainres.2004.09.059 15567342

[B31] Guo H. F. Tian J. Wang X. Fang Y. Hou Y. Han J. (1996). Brain substrates activated by electroacupuncture of different frequencies (I): comparative study on the expression of oncogene c-fos and genes coding for three opioid peptides. Mol. Brain Res. 43, 157–166. 10.1016/s0169-328x(96)00170-2 9037529

[B36] Han J.-S. (1995). Cholecystokinin octapeptide (CCK-8): a negative feedback control mechanism for opioid analgesia. Prog. Brain Res. 105, 263–271. 10.1016/s0079-6123(08)63303-8 7568886

[B37] Han J.-S. (2003). Acupuncture: neuropeptide release produced by electrical stimulation of different frequencies. Trends Neurosci. 26, 17–22. 10.1016/s0166-2236(02)00006-1 12495858

[B38] Han J.-S. (2011). Acupuncture analgesia: areas of consensus and controversy. Pain 152, S41–S48. 10.1016/j.pain.2010.10.012 21078546

[B34] Han C. S. Chou P. H. Lu C. C. Lu L. H. Yang T. H. Jen M. F. (1979). The role of central 5-hydroxytryptamine in acupuncture analgesia. Sci. Sin. 22, 91–104. 312529

[B41] Han J.-S. Ding X.-Z. Fan S.-G. (1985). Is cholecystokinin octapeptide (CCK-8) a candidate for endogenous antiopioid substrates? Neuropeptides 5, 399–402. 10.1016/0143-4179(85)90038-1 4000412

[B35] Han J. S. Ding X. Z. Fan S. G. (1986). Cholecystokinin octapeptide (CCK-8): antagonism to electroacupuncture analgesia and a possible role in electroacupuncture tolerance. Pain 27, 101–115. 10.1016/0304-3959(86)90227-7 3491355

[B42] Han Z. Jiang Y.-H. Wan Y. Wang Y. Chang J.-K. Han J.-S. (1999). Endomorphin-1 mediates 2 Hz but not 100 Hz electroacupuncture analgesia in the rat. Neurosci. Lett. 274, 75–78. 10.1016/s0304-3940(99)00670-9 10553941

[B60] Han J. S. Li S. J. Tang J. (1981). Tolerance to electroacupuncture and its cross tolerance to morphine. Neuropharmacology 20, 593–596. 10.1016/0028-3908(81)90213-6 7195480

[B39] Han J.-S. Wang Q. (1991). Arcuate nucleus (ARH) and parabrachial nucleus (PBN) mediate low-and high-frequency electroacupuncture analgesia. Acupunct. Res. 8, 81–182.

[B40] Han J.-S. Xuan Y.-T. (2009). A mesolimbic neuronal loop of analgesia: I. Activation by morphine of a serotonergic pathway from periaqueductal gray to nucleus accumbens. Int. J. Neurosci. 29, 109–117. 10.3109/00207458608985641 3486166

[B45] He L. F. Dong W. Q. (1983). Activity of opioid peptidergic system in acupuncture analgesia. Acupunct. Electrother. Res. 8, 257–266. 10.3727/036012983816714885 6145301

[B43] He C. Han J. (1990). Influence of microinjection of dynorphin antibody into periaqueductal gray (PAG) on analgesia induced by electroacupuncture of different frequencies in rats. Zhen Ci Yan Jiu 15, 97–103. 1980431

[B46] He L. Wang M. Gao M. Zhou J. (1992). Expression of c-fos protein in serotonergic neurons of rat brainstem following electro-acupuncture. Acupunct. Electrother. Res. 17, 243–248. 10.3727/036012992816357620 1362034

[B44] He J. Yan J. Chang X. Liu J. Li J. Yi S.. (2006). Neurons in the NTS of rat response to gastric distention stimulation and acupuncture at body surface points. Am. J. Chin. Med. 34, 427–433. 10.1142/s0192415x06003965 16710892

[B47] Heinricher M. M. Morgan M. M. Tortorici V. Fields H. L. (1994). Disinhibition of off-cells and antinociception produced by an opioid action within the rostral ventromedial medulla. Neuroscience 63, 279–288. 10.1016/0306-4522(94)90022-1 7898652

[B48] Hirano T. Zeredo J. L. Kimoto M. Moritaka K. Nasution F. H. Toda K. (2008). Disinhibitory involvement of the anterior cingulate cortex in the descending antinociceptive effect induced by electroacupuncture stimulation in rats. Am. J. Chin. Med. 36, 569–577. 10.1142/s0192415x08005989 18543389

[B49] Holden J. E. Schwartz E. J. Proudfit H. K. (1999). Microinjection of morphine in the A7 catecholamine cell group produces opposing effects on nociception that are mediated by α_1_- and α_2_-adrenoceptors. Neuroscience 91, 979–990. 10.1016/s0306-4522(98)00673-3 10391476

[B52] Hu Z. L. (1979). A study on the histological structure of acupuncture points and types of fibers conveying needling sensation. Chin. Med. J. 92:233. 155514

[B50] Hu M.-L. Qiu Z.-Y. Hu K. Ding M.-X. (2016). Analgesic neural circuits are activated by electroacupuncture at two sets of acupoints. Evid. Based Complement. Alternat. Med. 2016:3840202. 10.1155/2016/3840202 27429635 PMC4939346

[B51] Hu M.-L. Zhu H.-M. Zhang Q.-L. Liu J.-J. Ding Y. Zhong J.-M.. (2017). Exploring the mechanisms of electroacupuncture-induced analgesia through RNA sequencing of the periaqueductal gray. Int. J. Mol. Sci. 19:E2. 10.3390/ijms19010002 29295561 PMC5795954

[B57] Huang Y.-J. Grau J. W. (2018). Ionic plasticity and pain: the loss of descending serotonergic fibers after spinal cord injury transforms how GABA affects pain. Exp. Neurol. 306, 105–116. 10.1016/j.expneurol.2018.05.002 29729247 PMC5994379

[B53] Huang C. Hu Z.-P. Jiang S.-Z. Li H.-T. Han J.-S. Wan Y. (2007). CCKB receptor antagonist L365,260 potentiates the efficacy to and reverses chronic tolerance to electroacupuncture-induced analgesia in mice. Brain Res. Bull. 71, 447–451. 10.1016/j.brainresbull.2006.11.008 17259012

[B54] Huang C. Li H.-T. Shi Y.-S. Han J.-S. Wan Y. (2004). Ketamine potentiates the effect of electroacupuncture on mechanical allodynia in a rat model of neuropathic pain. Neurosci. Lett. 368, 327–331. 10.1016/j.neulet.2004.07.073 15364421

[B55] Huang C. Long H. Shi Y.-S. Han J.-S. Wan Y. (2005). Ketamine enhances the efficacy to and delays the development of tolerance to electroacupuncture-induced antinociception in rats. Neurosci. Lett. 375, 138–142. 10.1016/j.neulet.2004.10.086 15670657

[B56] Huang C. Wang Y. Han J.-S. Wan Y. (2002). Characteristics of electroacupuncture-induced analgesia in mice: variation with strain, frequency, intensity and opioid involvement. Brain Res. 945, 20–25. 10.1016/s0006-8993(02)02503-9 12113947

[B58] Ikeda H. Takasu S. Murase K. (2014). Contribution of anterior cingulate cortex and descending pain inhibitory system to analgesic effect of lemon odor in mice. Mol. Pain 10:14. 10.1186/1744-8069-10-14 24555533 PMC3936890

[B59] Jiang S.-W. Lin Y.-W. Hsieh C.-L. (2018). Electroacupuncture at hua tuo jia ji acupoints reduced neuropathic pain and increased GABAA receptors in rat spinal cord. Evid. Based Complement. Alternat. Med. 2018:8041820. 10.1155/2018/8041820 30069227 PMC6057337

[B61] Johansen J. P. Fields H. L. Manning B. H. (2001). The affective component of pain in rodents: direct evidence for a contribution of the anterior cingulate cortex. Proc. Natl. Acad. Sci. U S A 98, 8077–8082. 10.1073/pnas.141218998 11416168 PMC35470

[B62] Jones S. L. (1991). “Descending noradrenergic influences on pain,” in Progress in Brain Research, eds Barnes C. D. Pompeiano O. (Amsterdam: Elsevier), 381–394. 10.1016/s0079-6123(08)63824-8 1813927

[B63] Kang S.-Y. Kim C.-Y. Roh D.-H. Yoon S.-Y. Park J.-H. Lee H.-J.. (2011). Chemical stimulation of the ST36 acupoint reduces both formalin-induced nociceptive behaviors and spinal astrocyte activation *via* spinal α-2 adrenoceptors. Brain Res. Bull. 86, 412–421. 10.1016/j.brainresbull.2011.08.012 21889580

[B64] Kawakita K. Funakoshi M. (1982). Suppression of the jaw-opening reflex by conditioning a-delta fiber stimulation and electroacupuncture in the rat. Exp. Neurol. 78, 461–465. 10.1016/0014-4886(82)90063-2 7140908

[B67] Kim J. H. Kim H. Y. Chung K. Chung J. M. (2011). Electroacupuncture reduces the evoked responses of the spinal dorsal horn neurons in ankle-sprained rats. J. Neurophysiol. 105, 2050–2057. 10.1152/jn.00853.2010 21389301 PMC3094162

[B70] Kim W. Kim S. K. Min B.-I. (2013). Mechanisms of electroacupuncture-induced analgesia on neuropathic pain in animal model. Evid. Based Complement. Alternat. Med. 2013:436913. 10.1155/2013/436913 23983779 PMC3747484

[B65] Kim H. Y. Koo S. T. Kim J. H. An K. Chung K. Chung J. M. (2013). Electroacupuncture analgesia in rat ankle sprain pain model: neural mechanisms. Neurol. Res. 32, 10–17. 10.1179/016164109x12537002793689 20034438

[B66] Kim H. Kwon Y. Han H. Yang I. Beitz A. And Lee J. (2005). Antinociceptive mechanisms associated with diluted bee venom acupuncture (apipuncture) in the rat formalin test: involvement of descending adrenergic and serotonergic pathways. Pharmacol. Res. 51, 183–188. 10.1016/j.phrs.2004.07.011 15629266

[B68] Kim S. K. Min B.-I. Kim J. H. Hwang B. G. Yoo G. Y. Park D. S. (2005a). Effects of α1- and α2-adrenoreceptor antagonists on cold allodynia in a rat tail model of neuropathic pain. Brain Res. 1039, 207–210. 10.1016/j.brainres.2005.01.051 15781064

[B69] Kim S. K. Park J. H. Bae S. J. Kim J. H. Hwang B. G. Min B.-I.. (2005b). Effects of electroacupuncture on cold allodynia in a rat model of neuropathic pain: mediation by spinal adrenergic and serotonergic receptors. Exp. Neurol. 195, 430–436. 10.1016/j.expneurol.2005.06.018 16054138

[B71] Koo S. T. Lim K. S. Chung K. Ju H. Chung J. M. (2008). Electroacupuncture-induced analgesia in a rat model of ankle sprain pain is mediated by spinal α-adrenoceptors. Pain 135, 11–19. 10.1016/j.pain.2007.04.034 17537577 PMC2268107

[B72] Kwon Y.-B. Kang M.-S. Ahn C.-J. Han H.-J. Ahn B.-C. Lee J.-H. (2000). Effect of high or low frequency electroacupuncture on the cellular actitivy of catecholaminergic neurons in the brain stem. Acupunct. Electrother. Res. 25, 27–36. 10.3727/036012900816356235 10830973

[B73] Kwon Y.-B. Kang M.-S. Han H.-J. Beitz A. J. Lee J.-H. (2001). Visceral antinociception produced by bee venom stimulation of the Zhongwan acupuncture point in mice: role of α2 adrenoceptors. Neurosci. Lett. 308, 133–137. 10.1016/s0304-3940(01)01989-9 11457577

[B74] Kwon Y.-B. Yoon S.-Y. Kim H.-W. Roh D.-H. Kang S. Y. Ryu Y.-H.. (2006). Substantial role of locus coeruleus-noradrenergic activation and capsaicin-insensitive primary afferent fibers in bee venom’s anti-inflammatory effect. Neurosci. Res. 55, 197–203. 10.1016/j.neures.2006.03.003 16621078

[B75] Lai H.-C. Lin Y.-W. Hsieh C.-L. (2019). Acupuncture-analgesia-mediated alleviation of central sensitization. Evid. Based Complement. Alternat. Med. 2019:6173412. 10.1155/2019/6173412 30984277 PMC6431485

[B76] Lao L. Zhang R.-X. Zhang G. Wang X. Berman B. M. Ren K. (2004). A parametric study of electroacupuncture on persistent hyperalgesia and Fos protein expression in rats. Brain Res. 1020, 18–29. 10.1016/j.brainres.2004.01.092 15312783

[B77] Lapeyre S. Mauborgne A. Becker C. Benoliel J. J. Cesselin F. Hamon M.. (2001). Subcutaneous formalin enhances outflow of met-enkephalin- and cholecystokinin-like materials in the rat nucleus accumbens. Naunyn Schmiedebergs. Arch. Pharmacol. 363, 399–406. 10.1007/s002100000377 11330333

[B78] Lee J. H. Beitz A. J. (1993). The distribution of brain-stem and spinal cord nuclei associated with different frequencies of electroacupuncture analgesia. Pain 52, 11–28. 10.1016/0304-3959(93)90109-3 8446432

[B79] Lee J. H. Go D. Kim W. Lee G. Bae H. Quan F. S.. (2016). Involvement of spinal muscarinic and serotonergic receptors in the anti-allodynic effect of electroacupuncture in rats with oxaliplatin-induced neuropathic pain. Korean J. Physiol. Pharmacol. 20, 407–414. 10.4196/kjpp.2016.20.4.407 27382357 PMC4930909

[B80] Lei L.-G. Zhang Y.-Q. Zhao Z.-Q. (2004). Pain-related aversion and Fos expression in the central nervous system in rats. Neuroreport 15, 67–71. 10.1097/00001756-200401190-00014 15106833

[B81] Leung L. (2012). Neurophysiological basis of acupuncture-induced analgesia—an updated review. J. Acupunct. Meridian Stud. 5, 261–270. 10.1016/j.jams.2012.07.017 23265077

[B82] Lewis J. W. Baldrighi G. Akil H. (1987). A possible interface between autonomic function and pain control: opioid analgesia and the nucleus tractus solitarius. Brain Res. 424, 65–70. 10.1016/0006-8993(87)91193-0 3319042

[B84] Li S. J. Tang J. Han J. S. (1982). The implication of central serotonin in electro-acupuncture tolerance in the rat. Sci. Sin. B 25, 620–629. 6180474

[B83] Li A. Wang Y. Xin J. Lao L. Ren K. Berman B. M.. (2007). Electroacupuncture suppresses hyperalgesia and spinal Fos expression by activating the descending inhibitory system. Brain Res. 1186, 171–179. 10.1016/j.brainres.2007.10.022 18001697 PMC2200297

[B87] Liu X. (1996). The modulation of cerebral cortex and subcortical nuclei on NRM and their role in acupuncture analgesia. Zhen Ci Yan Jiu 21, 4–11. 9387347

[B85] Liu J.-L. Chen S.-P. Gao Y.-H. Meng F.-Y. Wang S.-B. Wang J.-Y. (2010). Effects of repeated electroacupuncture on β-endorphin and adrencorticotropic hormone levels in the hypothalamus and pituitary in rats with chronic pain and ovariectomy. Chin. J. Integr. Med. 16, 315–323. 10.1007/s11655-010-0503-3 20697942

[B86] Liu K. Gao X.-Y. Li L. Ben H. Qin Q.-G. Zhao Y.-X.. (2014). Neurons in the nucleus tractus solitarius mediate the acupuncture analgesia in visceral pain rats. Auton. Neurosci. 186, 91–94. 10.1016/j.autneu.2014.08.004 25204607

[B88] Liu X. Zhu B. Zhang S. (1986). Relationship between electroacupuncture analgesia and descending pain inhibitory mechanism of nucleus raphe magnus. Pain 24, 383–396. 10.1016/0304-3959(86)90124-7 3485785

[B91] Ma S.-X. (2004). Neurobiology of acupuncture: toward CAM. Evid. Based Complement. Alternat. Med. 1, 41–47. 10.1093/ecam/neh017 15257325 PMC442119

[B90] Ma Q. P. Han J. S. (1991). Neurochemical studies on the mesolimbic circuitry of antinociception. Brain Res. 566, 95–102. 10.1016/0006-8993(91)91685-t 1814560

[B89] Ma F. Xie H. Dong Z.-Q. Wang Y.-Q. Wu G.-C. (2004). Effects of electroacupuncture on orphanin FQ immunoreactivity and preproorphanin FQ mRNA in nucleus of raphe magnus in the neuropathic pain rats. Brain Res. Bull. 63, 509–513. 10.1016/j.brainresbull.2004.04.011 15249116

[B92] Migita K. Matsuzaki Y. Koga K. Matsumoto T. Mishima K. Hara S.. (2018). Involvement of GABAB receptor in the antihypersensitive effect in anterior cingulate cortex of partial sciatic nerve ligation model. J. Pharmacol. Sci. 137, 233–236. 10.1016/j.jphs.2018.05.009 30078433

[B93] Millan M. J. (1997). “The role of descending noradrenergic and serotoninergic pathways in the modulation of nociception: focus on receptor multiplicity,” in The Pharmacology of Pain Handbook of Experimental Pharmacology, eds Dickenson A. Besson J. M. (Berlin: Springer), 385–446.

[B94] Millan M. J. (2002). Descending control of pain. Prog. Neurobiol. 66, 355–474. 10.1016/S0301-0082(02)00009-6 12034378

[B95] Moran T. H. Schwartz G. J. (1994). Neurobiology of cholecystokinin. Crit. Rev. Neurobiol. 9, 1–28. 8828002

[B96] Niddam D. M. Chan R.-C. Lee S.-H. Yeh T.-C. Hsieh J.-C. (2007). Central modulation of pain evoked from myofascial trigger point. Clin. J. Pain 23, 440–448. 10.1097/AJP.0b013e318058accb 17515743

[B97] Noble F. Blommaert A. Fournié-Zaluski M.-C. Roques B. P. (1995). A selective CCKB receptor antagonist potentiates μ-, but not δ-opioid receptor-mediated antinociception in the formalin test. Eur. J. Pharmacol. 273, 145–151. 10.1016/0014-2999(94)00688-4 7737308

[B98] Pan B. Lopes J. M. C. Coimbra A. (1999). Central afferent pathways conveying nociceptive input to the hypothalamic paraventricular nucleus as revealed by a combination of retrograde labeling and c-fos activation. J. Comp. Neurol. 413, 129–145. 10.1002/(sici)1096-9861(19991011)413:1<129::aid-cne9>3.3.co;2-h 10464375

[B100] Park J.-H. Han J.-B. Kim S. K. Park J. H. Go D.-H. Sun B.. (2010). Spinal GABA receptors mediate the suppressive effect of electroacupuncture on cold allodynia in rats. Brain Res. 1322, 24–29. 10.1016/j.brainres.2010.02.001 20138846

[B99] Park D. S. Seo B. K. Baek Y. H. (2012). Analgesic effect of electroacupuncture on inflammatory pain in collagen-induced arthritis rats: mediation by α2- and β-adrenoceptors. Rheumatol. Int. 33, 309–314. 10.1007/s00296-012-2369-5 22441959

[B101] Pedrón V. T. Varani A. P. Bettler B. Balerio G. N. (2019). GABAB receptors modulate morphine antinociception: pharmacological and genetic approaches. Pharmacol. Biochem. Behav. 180, 11–21. 10.1016/j.pbb.2019.02.015 30851293

[B102] Pertovaara A. Almeida A. (2006). Chapter 13 descending inhibitory systems. Handb. Clin. Neurol. 81, 179–192. 10.1016/s0072-9752(06)80017-5 18808835

[B103] Pomeranz B. Berman B. (2003). “Scientific basis of acupuncture,” in Basics of Acupuncture, eds Stux G. Berman B. Pomeranz B. (Berlin: Springer), 7–86.

[B104] Porreca F. Ossipov M. H. Gebhart G. F. (2002). Chronic pain and medullary descending facilitation. Trends Neurosci. 25, 319–325. 10.1016/s0166-2236(02)02157-4 12086751

[B105] Proudfit H. K. (1980). Effects of raphe magnus and raphe pallidus lesions on morphine-induced analgesia and spinal cord monoamines. Pharmacol. Biochem. Behav. 13, 705–714. 10.1016/0091-3057(80)90015-5 7443740

[B106] Pu S.-F. Zhuang H.-X. Han J.-S. (1994). Cholecystokinin octapeptide (CCK-8) antagonizes morphine analgesia in nucleus accumbens of the rat *via* the CCK-B receptor. Brain Res. 657, 159–164. 10.1016/0006-8993(94)90963-6 7820614

[B107] Qiao L. N. Liu J.-L. Tan L. H. Yang H.-L. Zhai X. Yang Y. S. (2017). Effect of electroacupuncture on thermal pain threshold and expression of calcitonin-gene related peptide, substance P and γ-aminobutyric acid in the cervical dorsal root ganglion of rats with incisional neck pain. Acupunct. Med. 35, 276–283. 10.1136/acupmed-2016-011177 28600329 PMC5561363

[B108] Qiao L. N. Yang Y. S. Liu J.-L. Zhu J. Tan L. H. Shi Y. N.. (2019). Contribution of GABAergic modulation in DRGs to electroacupuncture analgesia in incisional neck pain rats. J. Pain Res. 12, 405–416. 10.2147/jpr.s180165 30705606 PMC6342219

[B109] Qiu Z.-Y. Ding Y. Cui L.-Y. Hu M.-L. Ding M.-X. (2015). The expression patterns of c-fos and c-jun induced by different frequencies of electroacupuncture in the brain. Evid. Based Complement. Alternat. Med. 2015:343682. 10.1155/2015/343682 26491460 PMC4603316

[B110] Radhakrishnan R. King E. W. Dickman J. K. Herold C. A. Johnston N. F. Spurgin M. L.. (2003). Spinal 5-HT2 and 5-HT3 receptors mediate low, but not high, frequency TENS-induced antihyperalgesia in rats. Pain 105, 205–213. 10.1016/s0304-3959(03)00207-0 14499437 PMC2746627

[B111] Rainville P. Duncan G. H. Price D. D. Carrier B. Bushnell M. C. (1997). Pain affect encoded in human anterior cingulate but not somatosensory cortex. Science 277, 968–971. 10.1126/science.277.5328.968 9252330

[B112] Rehfeld J. F. (1980). Cholecystokinin. Trends Neurosci. 3, 65–67. 10.1016/0166-2236(80)90025-9

[B113] Ren K. Randich A. Gebhart G. F. (1990). Modulation of spinal nociceptive transmission from nuclei tractus solitarii: a relay for effects of vagal afferent stimulation. J. Neurophysiol. 63, 971–986. 10.1152/jn.1990.63.5.971 1972739

[B114] Roh D.-H. Kwon Y.-B. Kim H.-W. Ham T.-W. Yoon S.-Y. Kang S. Y.. (2004). Acupoint stimulation with diluted bee venom (apipuncture) alleviates thermal hyperalgesia in a rodent neuropathic pain model: involvement of spinal α2-adrenoceptors. J. Pain 5, 297–303. 10.1016/j.jpain.2004.05.003 15336634

[B115] Saadé N. E. Barchini J. Tchachaghian S. Chamaa F. Jabbur S. J. Song Z.. (2014). The role of the dorsolateral funiculi in the pain relieving effect of spinal cord stimulation: a study in a rat model of neuropathic pain. Exp. Brain Res. 233, 1041–1052. 10.1007/s00221-014-4180-x 25537469

[B116] Sandkühler J. (1996). The organization and function of endogenous antinociceptive systems. Prog. Neurobiol. 50, 49–81. 10.1016/s0301-0082(96)00031-7 8931107

[B117] Seo B. K. Sung W.-S. Park Y.-C. Baek Y. H. (2016). The electroacupuncture-induced analgesic effect mediated by 5-HT_1_, 5-HT_3_ receptor and muscarinic cholinergic receptors in rat model of collagenase-induced osteoarthritis. BMC Complement. Altern. Med. 16:212. 10.1186/s12906-016-1204-z 27411565 PMC4943008

[B118] Silva J. R. T. Silva M. L. Prado W. A. (2011). Analgesia induced by 2- or 100-Hz electroacupuncture in the rat tail-flick test depends on the activation of different descending pain inhibitory mechanisms. J. Pain 12, 51–60. 10.1016/j.jpain.2010.04.008 20554480

[B119] Sluka K. A. Lisi T. L. Westlund K. N. (2006). Increased release of serotonin in the spinal cord during low, but not high, frequency transcutaneous electric nerve stimulation in rats with joint inflammation. Arch. Phys. Med. Rehabil. 87, 1137–1140. 10.1016/j.apmr.2006.04.023 16876561 PMC2746636

[B120] Song Z. Ansah O. B. Meyerson B. A. Pertovaara A. Linderoth B. (2013). The rostroventromedial medulla is engaged in the effects of spinal cord stimulation in a rodent model of neuropathic pain. Neuroscience 247, 134–144. 10.1016/j.neuroscience.2013.05.027 23711584

[B122] Takagi J. Sawada T. Yonehara N. (1996). A possible involvement of monoaminergic and opioidergic systems in the analgesia induced by electro-acupuncture in rabbits. Jpn. J. Pharmacol. 70, 73–80. 10.1254/jjp.70.73 8822091

[B121] Takagi J. Yonehara N. (1998). Serotonin receptor subtypes involved in modulation of electrical acupuncture. Jpn. J. Pharmacol. 78, 511–514. 10.1254/jjp.78.511 9920210

[B123] Takeshige C. Nakamura A. Asamoto S. Arai T. (1992a). Positive feedback action of pituitary β-endorphin on acupuncture analgesia afferent pathway. Brain Res. Bull. 29, 37–44. 10.1016/0361-9230(92)90006-j 1324098

[B124] Takeshige C. Sato T. Mera T. Hisamitsu T. Fang J. (1992b). Descending pain inhibitory system involved in acupuncture analgesia. Brain Res. Bull. 29, 617–634. 10.1016/0361-9230(92)90131-g 1422859

[B125] Takeshige C. Tsuchiya M. Guo S. Y. Sato T. (1991). Dopaminergic transmission in the hypothalamic arcuate nucleus to produce acupuncture analgesia in correlation with the pituitary gland. Brain Res. Bull. 26, 113–122. 10.1016/0361-9230(91)90195-p 1849781

[B127] Tang N.-M. Dong H.-W. Wang X.-M. Tsui Z.-C. Han J.-S. (1997). Cholecystokinin antisense RNA increases the analgesic effect induced by electroacupuncture or low dose morphine: conversion of low responder rats into high responders. Pain 71, 71–80. 10.1016/s0304-3959(97)03341-1 9200176

[B126] Tang J. Li S. J. Xie C. W. Han J. S. (1981). “The role of central 5-hydroxytryptamine in acupuncture analgesia and acupuncture tolerance,” in Advances in Endogenous and Exogenous Opioids, eds Takagi H. Simon E. J. (Kyoto: Elsevier), 300–302. 10.1016/B978-0-444-80402-0.50102-9

[B128] Tian J.-H. Xu W. Zhang W. Fang Y. Grisel J. E. Mogil J. S.. (1997). Involvement of endogenous Orphanin FQ in electroacupuncture-induced analgesia. Neuroreport 8, 497–500. 10.1097/00001756-199701200-00024 9080436

[B129] Toda K. (2002). Afferent nerve characteristics during acupuncture stimulation. Int. Congress Ser. 1238, 49–61. 10.1016/s0531-5131(02)00421-1

[B130] Treede R.-D. Rief W. Barke A. Aziz Q. Bennett M. I. Benoliel R.. (2019). Chronic pain as a symptom or a disease: the IASP Classification of Chronic Pain for the: international classification of diseases (ICD-11). Pain 160, 19–27. 10.1097/j.pain.0000000000001384 30586067

[B131] Tsuda M. Koga K. Chen T. Zhuo M. (2017). Neuronal and microglial mechanisms for neuropathic pain in the spinal dorsal horn and anterior cingulate cortex. J. Neurochem. 141, 486–498. 10.1111/jnc.14001 28251660

[B132] Vanegas H. Schaible H.-G. (2004). Descending control of persistent pain: inhibitory or facilitatory? Neuroimage 46, 295–309. 10.1016/j.brainresrev.2004.07.004 15571771

[B133] Wall P. D. (1967). The laminar organization of dorsal horn and effects of descending impulses. J. Physiol. 188, 403–423. 10.1113/jphysiol.1967.sp008146 6032207 PMC1396017

[B135] Wang X. Shi H. Shang H. He W. Chen S. Litscher G.. (2013). Effect of electroacupuncture at ST36 on gastric-related neurons in spinal dorsal horn and nucleus tractus solitarius. Evid. Based Complement. Alternat. Med. 2013:912898. 10.1155/2013/912898 24191172 PMC3804039

[B136] Wang X.-J. Wang X.-H. Han J.-S. (1990). Cholecystokinin octapeptide antagonized opioid analgesia mediated by μ- and κ- but not δ-receptors in the spinal cord of the rat. Brain Res. 523, 5–10. 10.1016/0006-8993(90)91629-u 1976419

[B134] Wang H. Zhu C. B. Cao X. D. Wu G. C. (1998). Effect of orphanin FQ on acupuncture analgesia and noxious stimulation in the periaqueductal gray. Sheng Li Xue Bao 50, 263–267. 11324565

[B137] Wiesenfeld-Hallin Z. Xu X.-J. (1996). The role of cholecystokinin in nociception, neuropathic pain and opiate tolerance. Regul. Pept. 65, 23–28. 10.1016/0167-0115(96)00068-7 8876032

[B138] Winters B. L. Christie M. J. Vaughan C. W. (2019). Electrophysiological actions of N/OFQ. Handb. Exp. Pharmacol. 254, 91–130. 10.1007/164_2019_205 30838458

[B139] Wu M.-T. Hsieh J.-C. Xiong J. Yang C.-F. Pan H.-B. Chen Y.-C. I.. (1999). Central nervous pathway for acupuncture stimulation: localization of processing with functional MR imaging of the brain—preliminary experience. Radiology 212, 133–141. 10.1148/radiology.212.1.r99jl04133 10405732

[B140] Wyss J. M. Sripanidkulchai K. (1984). The topography of the mesencephalic and pontine projections from the cingulate cortex of the rat. Brain Res. 293, 1–15. 10.1016/0006-8993(84)91448-3 6704709

[B141] Xiang L. Zhang S. Weidong X. Xiaoling H. (1991). The influence of transection of dorsal lateral fasciculus (DLF) on the effects of SMII stimulation on nucleus raphe magnus (MRM). Acupunct. Res. 224–225. 10.13702/j.1000-0607.1991.z1.067

[B142] Xu W. Qiu X. C. Han J. S. (1994). Serotonin receptor subtypes in spinal antinociception in the rat. J. Pharmacol. Exp. Ther. 269, 1182–1189. 8014862

[B143] Xuan Y. T. Shi Y. S. Zhou Z. F. Han J. S. (1986). Studies on the mesolimbic loop of antinociception—II. A serotonin-enkephalin interaction in the nucleus accumbens. Neuroscience 19, 403–409. 10.1016/0306-4522(86)90270-8 3022186

[B144] Yaksh T. L. (1985). Pharmacology of spinal adrenergic systems which modulate spinal nociceptive processing. Pharmacol. Biochem. Behav. 22, 845–858. 10.1016/0091-3057(85)90537-4 2861606

[B145] Yaksh T. L. Rudy T. A. (1977). Narcotic analgetics: CNS sites and mechanisms of action as revealed by intracerebral injection techniques. Pain 4, 299–359. 10.1016/0304-3959(77)90145-2 25403

[B146] Yaksh T. L. Tyce G. M. (1979). Microinjection of morphine into the periaqueductal gray evokes the release of serotonin from spinal cord. Brain Res. 171, 176–181. 10.1016/0006-8993(79)90747-9 466437

[B147] Yan B. Li K. Xu J. Wang W. Li K. Li K.. (2005). Acupoint-specific fMRI patterns in human brain. Neurosci. Lett. 383, 236–240. 10.1016/j.neulet.2005.04.021 15876491

[B148] Yan L.-P. Wu X.-T. Yin Z.-Y. Ma C. (2011). Effect of electroacupuncture on the levels of amino acid neurotransmitters in the spinal cord in rats with chronic constrictive injury. Zhen Ci Yan Jiu 36, 353–6379. 10.1111/j.1600-0714.2011.01024.x 22073887

[B149] Yi M. Zhang H. Lao L. Xing G.-G. Wan Y. (2011). Anterior cingulate cortex is crucial for contra- but not ipsi-lateral electro-acupuncture in the formalin-induced inflammatory pain model of rats. Mol. Pain 7:61. 10.1186/1744-8069-7-61 21854647 PMC3170225

[B150] Yin Q. H. Mao J. R. Guo S. Y. (1988). Changes of reactions of neurones in dorsal raphe nucleus and locus coeruleus to electroacupuncture by hypothalamic arcuate nucleus stimulation. Funct. Neurol. 3, 263–273. 3192102

[B151] Yoo Y.-C. Oh J. H. Kwon T. D. Lee Y. K. Bai S. J. (2011). Analgesic mechanism of electroacupuncture in an arthritic pain model of rats: a neurotransmitter study. Yonsei Med. J. 52, 1016–1021. 10.3349/ymj.2011.52.6.1016 22028168 PMC3220264

[B152] Yoon S.-Y. Roh D.-H. Kwon Y.-B. Kim H.-W. Seo H.-S. Han H.-J.. (2009). Acupoint stimulation with diluted bee venom (apipuncture) potentiates the analgesic effect of intrathecal clonidine in the rodent formalin test and in a neuropathic pain model. J. Pain 10, 253–263. 10.1016/j.jpain.2008.09.002 19010737

[B153] Yu J.-S. Zeng B.-Y. Hsieh C.-L. (2013). Acupuncture Stimulation and Neuroendocrine Regulation. 1st Edn. Elsevier Inc. 10.1016/B978-0-12-411545-3.00006-7 24215920

[B154] Yuan X.-C. Zhu B. Jing X.-H. Xiong L.-Z. Wu C.-H. Gao F.. (2018). Electroacupuncture potentiates cannabinoid receptor-mediated descending inhibitory control in a mouse model of knee osteoarthritis. Front. Mol. Neurosci. 11:112. 10.3389/fnmol.2018.00112 29681797 PMC5897736

[B155] Zhang H. Chen X. Zhang C. Zhang R. Lao L. Wan Y.. (2013). Comparison of electroacupuncture in restrained and unrestrained rat models. Evid. Based Complement. Alternat. Med. 2013:404956. 10.1155/2013/404956 23737830 PMC3666275

[B160] Zhang W.-T. Jin Z. Luo F. Zhang L. Zeng Y.-W. Han J.-S. (2004). Evidence from brain imaging with fMRI supporting functional specificity of acupoints in humans. Neurosci. Lett. 354, 50–53. 10.1016/j.neulet.2003.09.080 14698480

[B161] Zhang Y. Li A. Lao L. Xin J. Ren K. Berman B. M.. (2011a). Rostral ventromedial medulla μ, but not κ, opioid receptors are involved in electroacupuncture anti-hyperalgesia in an inflammatory pain rat model. Brain Res. 1395, 38–45. 10.1016/j.brainres.2011.04.037 21565329 PMC3105222

[B162] Zhang Y. Li A. Xin J. Lao L. Ren K. Berman B. M.. (2011b). Involvement of spinal serotonin receptors in electroacupuncture anti-hyperalgesia in an inflammatory pain rat model. Neurochem. Res. 36, 1785–1792. 10.1007/s11064-011-0495-1 21556842 PMC3581079

[B159] Zhang S. Liu Y. Ye Y. Wang X.-R. Lin L.-T. Xiao L.-Y.. (2018). Bee venom therapy: potential mechanisms and therapeutic applications. Toxicon 148, 64–73. 10.1016/j.toxicon.2018.04.012 29654868

[B157] Zhang Q. Tan Y.-Y. Liu X.-H. Yao F.-R. Cao D.-Y. (2018). Electroacupuncture improves baroreflex and γ-aminobutyric acid type b receptor-mediated responses in the nucleus tractus solitarii of hypertensive rats. Neural Plast. 2018:8919347. 10.1155/2018/8919347 30363902 PMC6186317

[B158] Zhang R. X. Wang R. Chen J. Y. Qiao J. T. (1994). Effects of descending inhibitory systems on the c-Fos expression in the rat spinal cord during formalin-induced noxious stimulation. Neuroscience 58, 299–304. 10.1016/0306-4522(94)90036-1 8152541

[B163] Zhang Y. Zhang R. X. Zhang M. Shen X. Y. Li A. Xin J.. (2012). Electroacupuncture inhibition of hyperalgesia in an inflammatory pain rat model: involvement of distinct spinal serotonin and norepinephrine receptor subtypes. Br. J. Anaesth. 109, 245–252. 10.1093/bja/aes136 22628394 PMC3393079

[B156] Zhang L. Zhang Y. Zhao Z.-Q. (2005). Anterior cingulate cortex contributes to the descending facilitatory modulation of pain *via* dorsal reticular nucleus. Eur. J. Neurosci. 22, 1141–1148. 10.1111/j.1460-9568.2005.04302.x 16176356

[B164] Zhao Z.-Q. (2008). Neural mechanism underlying acupuncture analgesia. Prog. Neurobiol. 85, 355–375. 10.1016/j.pneurobio.2008.05.004 18582529

[B166] Zhou Y. Sun Y. H. Shen J. M. Han J. S. (1993). Increased release of immunoreactive CCK-8 by electroacupuncture and enhancement of electroacupuncture analgesia by CCK-B antagonist in rat spinal cord. Neuropeptides 24, 139–144. 10.1016/0143-4179(93)90077-n 8474632

[B167] Zhou Y. Sun Y. H. Zhang Z. W. Han J. S. (1992). Accelerated expression of cholecystokinin gene in the brain of rats rendered tolerant to morphine. Neuroreport 3, 1121–1123. 10.1097/00001756-199212000-00022 1283533

[B165] Zhou M. M. Yu C. X. Wang M. Z. Cao X. D. Wu G. C. (2001). Alteration of orphanin FQ immunoreactivity and ppOFQ mRNA by combination of melatonin with electroacupuncture. Acupunct. Electrother. Res. 26, 49–58. 10.3727/036012901816356018 11394493

